# Evaluating the Efficacy of L-N-acetylcysteine and Dexamethasone in Combination to Provide Otoprotection for Electrode Insertion Trauma

**DOI:** 10.3390/jcm9030716

**Published:** 2020-03-06

**Authors:** Adrien A. Eshraghi, David Shahal, Camron Davies, Jeenu Mittal, Viraj Shah, Erdogan Bulut, Carolyn Garnham, Priyanka Sinha, Dibyanshi Mishra, Hannah Marwede, Rahul Mittal

**Affiliations:** 1 Department of Otolaryngology, Cochlear Implant and Hearing Research Laboratory, University of Miami Miller School of Medicine, Miami, FL 33136, USA; dxs1298@miami.edu (D.S.); cdavi191@med.fiu.edu (C.D.); j.mittal@med.miami.edu (J.M.); viraj.shah@med.miami.edu (V.S.); exb832@miami.edu (E.B.); priyankasinha.ras@gmail.com (P.S.); dxm1167@miami.edu (D.M.); hxm352@miami.edu (H.M.); r.mittal11@med.miami.edu (R.M.); 2 Department of Neurological Surgery, University of Miami Miller School of Medicine, Miami, FL 33136, USA; 3 Department of Biomedical Engineering, University of Miami, Coral Gables, FL 33143, USA; 4 MED-EL Hearing Implants, 6020 Innsbruck, Austria; j.mittal@miami.edu

**Keywords:** electrode insertion trauma, residual hearing, L-N-acetylcysteine, dexamethasone, organ of Corti, oxidative stress, proinflammatory cytokines, nitric oxide

## Abstract

Background: Electrode insertion trauma (EIT) during cochlear implantation (CI) can cause loss of residual hearing. L-N-acetylcysteine (L-NAC) and dexamethasone (Dex) have been individually shown to provide otoprotection albeit at higher concentrations that may be associated with adverse effects. Objective/Aims: The aim of this study is to determine whether L-NAC and Dex could be combined to decrease their effective dosage. Materials and Methods: The organ of Corti (OC) explants were divided into various groups: 1) control; 2) EIT; 3) EIT treated with different concentrations of Dex; 4) EIT treated with different concentrations of L-NAC; 5) EIT treated with L-NAC and Dex in combination. Hair cell (HC) density, levels of oxidative stress, proinflammatory cytokines and nitric oxide (NO) was determined. Results: There was a significant loss of HCs in explants subjected to EIT compared to the control group. L-NAC and Dex in combination was able to provide significant otoprotection at lower concentrations compared to individual drugs. Conclusions and Significance: A combination containing L-NAC and Dex is effective in protecting sensory cells at lower protective doses than each compound separately. These compounds can be combined allowing a decrease of potential side effects of each compound and providing significant otoprotection for EIT.

## 1. Introduction

Cochlear implantations (CIs) have provided multiple benefits for patients, resulting in expanding indications over the years, in both adult and pediatric patients mainly with bilateral severe-to-profound sensorineural hearing loss [1,2]. According to National Institute on Deafness and Other Communication Disorders (NIDCD) statistical data from year 2012, more than 324,200 registered devices have been implanted worldwide [3]. In the United States, more than 58,000 devices have been implanted in adults and 38,000 in children [3]. CIs represent the current standard of care for auditory rehabilitation, reestablishing the function of the inner hair cells by converting the acoustic signal to electrical stimuli for activation of cochlear nerve fibers [3]. Children with deafness detected early after onset can reach near to normal language development when undergoing a prompt CI. The approximate cost for CI is around 60,000 USD that includes procedure, training and adjustments [3]. On the other hand, the estimates costs are higher than $1 million for special education and related services for a child less than three years old with untreated deafness. Improvement in speech processor and electrode technologies have helped these children to succeed in conventional classrooms. Hearing-impaired patients that previously used appropriate hearing aids with adequate amplification but little to no useful benefit can take advantage of CI technology, as positive outcomes from the procedure far outweigh the risks. These findings have led to higher motivation and realistic expectations from patients and their families.

One challenge of CI surgery is the preservation of residual hearing [4,5]. CI can induce electrode insertion trauma (EIT) that can result in damage to critical sensory structures of the inner ear including the modiolus, spiral lamina, spiral ligament, stria vascularis, and the organ of Corti (OC) during initial electrode insertion [6,7,8,9,10]. Moreover, this immediate structural damage is followed by an inflammatory response mediated by the ischemia-reperfusion injury, immune cell recruitment and the generation of reactive oxygen species (ROS) which ultimately lead to further structural damage and apoptosis of hair cells [8]. This process is chronically propagated by the foreign body reaction against the electrode array accompanied with fibrosis, neo-osteogenesis, and infiltration of free radical producing inflammatory cells such as mononuclear leukocytes and histiocytes [8]. As these mechanisms occur near vital structures such as OC, they can lead to hair cell death and result in residual hearing loss well after the initial traumatic event [7]. Therefore, while the direct trauma is an area of concern and should be improved by technological and surgical advances, the initiation of oxidative stress and inflammatory response may play a crucial role in loss of residual hearing. Otoprotective compounds such as L-N-acetylcysteine (L-NAC) and dexamethasone (Dex) targeting oxidative stress, inflammatory and apoptotic pathways hold a great potential in developing therapeutic strategies to prevent the loss of residual hearing post-CI. However, higher concentrations of these drugs are needed to effectively counteract these pathways that may be associated with adverse effects. Therefore, in this study we determined whether combining L-NAC and Dex reduces their effective dosage and provides otoprotection for EIT employing an ex vivo model [11].

## 2. Materials and Methods

This animal study was approved by the Animal Care and Use Committee of the University of Miami (Protocol number 18-117-LF; Date of approval 8/2/2018) and fully complies with the NIH guidelines for the care and use of laboratory animals.

### 2.1. Organ of Corti (OC) Dissections

OCs were dissected from three-day-old (P-3) Sprague-Dawley laboratory rats (Charles River Laboratories, Inc., Wilmington, MA) [11]. Rats were anesthetized in ice for 30 min. OC explants were harvested and placed in serum-free media consisting of Dulbecco’s modified Eagle’s medium (DMEM) supplemented with glucose (final conc. 6 g/L), N-1 supplement (1%), and penicillin G (500 U/mL). Explants were cultured at 37 °C in a 95% humidified atmosphere and 5% CO_2._ The explants were divided into eight groups: 1) control group—no trauma and no drug; 2) untreated EIT group; 3) EIT and treated with 5 µg/mL Dex; 4) EIT and treated with 10 µg/mL Dex; 5) EIT and treated with 20 µg/mL Dex; 6) EIT and treated with 2 mM L-NAC; 7) EIT and treated with 5 mM L-NAC; 8) EIT and treated with 10 µg/mL Dex and 2 mM L-NAC in combination. OCs in each group were cultured for 24 h. Nine explants were used for each group per experiment. Dex was purchased as a commercial solution from American Regent company. L-NAC was purchased from Sigma (St. Louis, MO, USA) and stock solutions were made in sterile water.

For EIT, a 0.28-mm diameter monofilament fishing line (Cajun Line; W.C. Bradley, Tulsa, OK, USA) was introduced three times through the small 0.35 mm diameter cochleostomy located next to the round window niche, in order to achieve a high angle and depth of insertion into the scala tympani, which varied between 110–150 degrees as described in detail in previous studies [11]. The time from the point of insertion of the fishing line to withdrawal was equally maintained for all insertions in experimental animals.

### 2.2. FITC-Phalloidin Staining

OCs were subjected to FITC-phalloidin staining to visualize HCs. OC explants were cultured for 24h and then fixed in 4% paraformaldehyde (PFA) [11]. After fixation, the explants were washed three times in PBS and incubated in 5% normal goat serum (Sigma Aldrich, St. Louis, MO, USA) and 1% Triton X-100 (Fluka, St. Louis, MO, USA) in PBS for 90 min at 25 °C Then the samples were washed three times with PBS and incubated with FITC-labeled phalloidin (Sigma Aldrich, St. Louis, MO, USA) for 90 min at 25 °C An additional three washes with PBS was performed and then the samples were mounted with mounting medium having 4′,6-diamidino-2-phenylindole (DAPI) (Vector laboratories, Burlingame, CA, USA) cover slipped, and viewed under a confocal Zeiss Axiovert 700 microscope (Carl Zeiss Microimaging, LLC; Thornwood, New York, NY, USA). Stereocilia bundles of HCs stained with phalloidin-FITC were recognized and used for HC counts. A HC was counted if it possessed an intact cuticular plate with an intact stereociliary bundle.

### 2.3. Oxidative Stress Determination

To determine the levels of oxidative stress, OCs were subjected to immunostaining with CellROX [11]. Samples were incubated with CellROX Deep Red (5 µM, Thermofisher Scientific, Waltham, MA, USA) at 37 °C for 30 min. OCs were then washed three times with PBS fixed in 4% paraformaldehyde and 1% methanol in 0.1 M PBS overnight. Samples were washed three times with PBS followed by incubation in 5% normal goat serum (Sigma Aldrich, St Louis, MO, USA) and 1% Triton X-100 (Fluka, St Louis, MO, USA) in PBS for 30 min. OCs were then incubated with FITC-labeled phalloidin for 45 min followed by washing with PBS and incubation with 600 nM 4′, 6-diamidino-2-phenylindole (DAPI) solution (Sigma Aldrich, St Louis, MO, USA) for 5 min. After washing, samples were transferred to a glass slide with a mounting medium, cover slipped, and viewed under a confocal Zeiss Axiovert 700 microscope (Carl Zeiss AG, Jena, Germany). ImageJ software was used for processing and analyzing the images. For quantification, red signal intensity was measured, and the background was subtracted. The size of region of interest (ROI) was the same for all images.

### 2.4. Determination of Cytokine Production

The levels of proinflammatory cytokines, TNF-α, IL-1β, and IL-6 were determined in explant homogenates using ELISA kits as per manufacturer’s instructions (Thermofisher Scientific, Waltham, MA, USA)

### 2.5. Measurement of Nitric Oxide (NO) Release

NO levels were determined in OC homogenates using commercially available kit (Abcam, Cambridge, MA, USA). Cochlear explants were homogenized in 100 μL of Nitrite Assay Buffer (provided in the kit), followed by centrifugation at 3000 rpm for 30 min at 4 °C The supernatant was collected, and NO concentration was quantitated using Griess reagent as per manufacturer’s instructions (Abcam, Cambridge, MA, USA).

### 2.6. Statistical Analysis

Two-way analysis of variance (ANOVA) test was used followed by a Bonferroni post-test for multiple comparisons. P values of less than 0.05 was considered significant. All calculations were performed using SPSS software version 24.

## 3. Results

### 3.1. Effect of L-NAC and Dex on Hair Cell Viability

**Figure 1:**
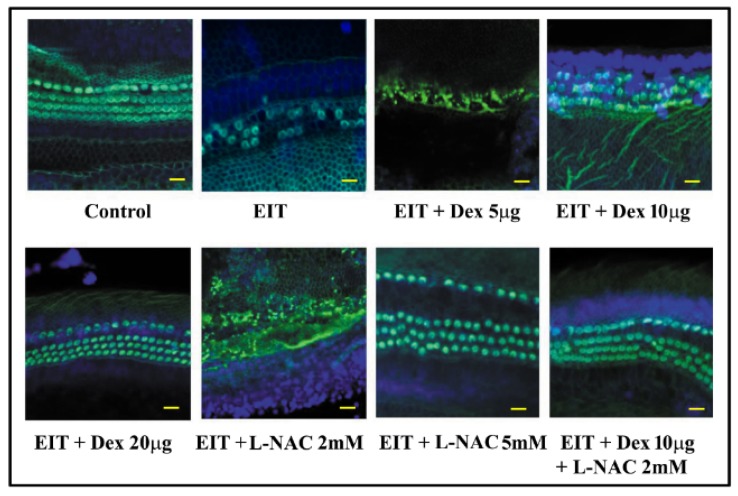
L-N-acetylcysteine (L-NAC) and dexamethasone (Dex) provides otoprotection against electrode insertion trauma (EIT). Organ of Corti (OC) explants were exposed to EIT alone or EIT and treated with L-NAC and Dex either individually or in combination. Samples were subjected to FITC-phalloidin staining to visualize hair cells (HCs). Results are representative of four independent experiments. Image represents middle + base area of explant. *n* = 9 OCs per group per experiment. Scale bars: 10 micrometers.

**Figure 2:**
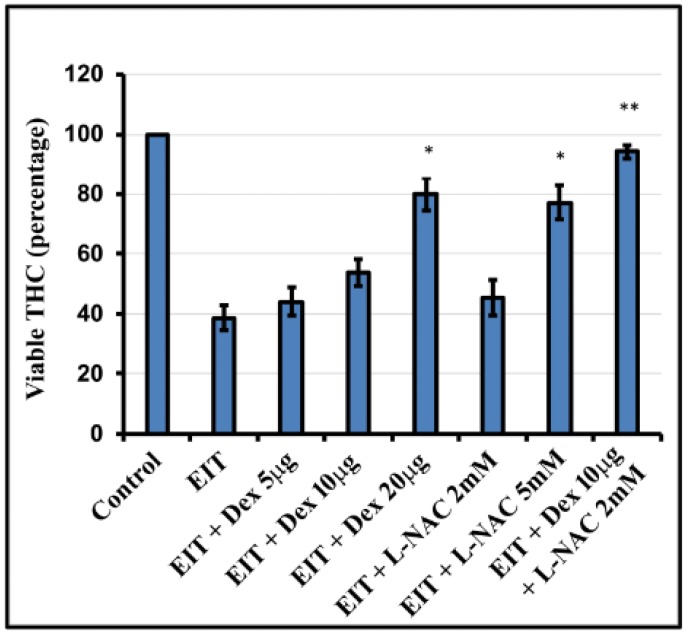
Hair cell quantification. Total hair cells (THCs) were counted based on FITC-phalloidin staining. EIT-exposed OC explants showed a significant decrease in the number of THC compared to control group. L-NAC and Dex in combination was able to significantly prevent EIT induced sensory cell loss at lower doses. Data are expressed as mean values ± SD and are representative of four independent experiments. * *P* < 0.05 or ** *P* < 0.001 compared to EIT group. *n* = 9 OCs per group per experiment.

There was a significant decrease in total hair cell count in the EIT explants compared with the control group (*P* < 0.001) (Figure 1 and Figure 2). We observed that individually, lower concentration of Dex and L-NAC provide only 50% protection against EIT whereas higher concentrations of 20 µg/mL Dex and 5 mM L-NAC provide efficient otoprotection. However, in combination lower concentrations of 10 µg/mL Dex + 2 mM L-NAC provide more than 90% otoprotection against HC loss suggesting additive or synergistic interaction between two compounds.

### 3.2. L-NAC and Dex Significantly Downregulate Oxidative Stress

**Figure 3:**
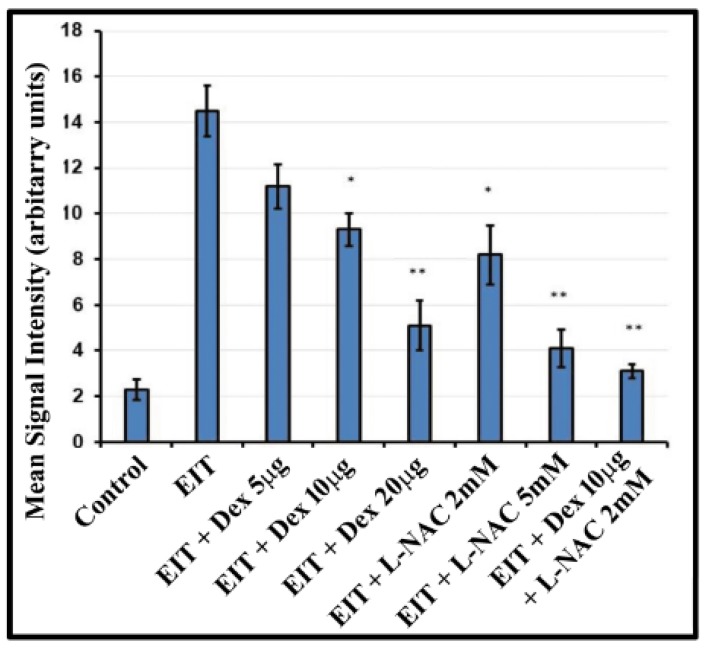
Oxidative stress in OC explants. Mean signal intensity for CellROX was calculated using Image J software. Data are expressed as mean values ± SD and is representative of four independent experiments. * *P* < 0.05 or ** *P* < 0.001 compared to EIT group. *n* = 9 OCs per group per experiment.

CellROX was used as a marker of oxidative stress. OC explants subjected to EIT showed strong immunolabeling for CellROX. Individually, higher concentrations of 20 µg/mL Dex and 5 mM L-NAC significantly reduced CellROX immunolabeling (*P* < 0.01) (Figure 3). However, treatment with lower concentrations of 10 µg/mL Dex + 2 mM L-NAC in combination was able to significantly decrease CellROX immunolabeling (*P* < 0.001). The mean signal intensity for CellROX immunostaining was significantly lower when OC explants were treated with 10 µg/mL Dex + 2 mM L-NAC (Figure 3).

### 3.3. L-NAC and Dex Significantly Decrease the Production of Proinflammatory Cytokines

**Figure 4:**
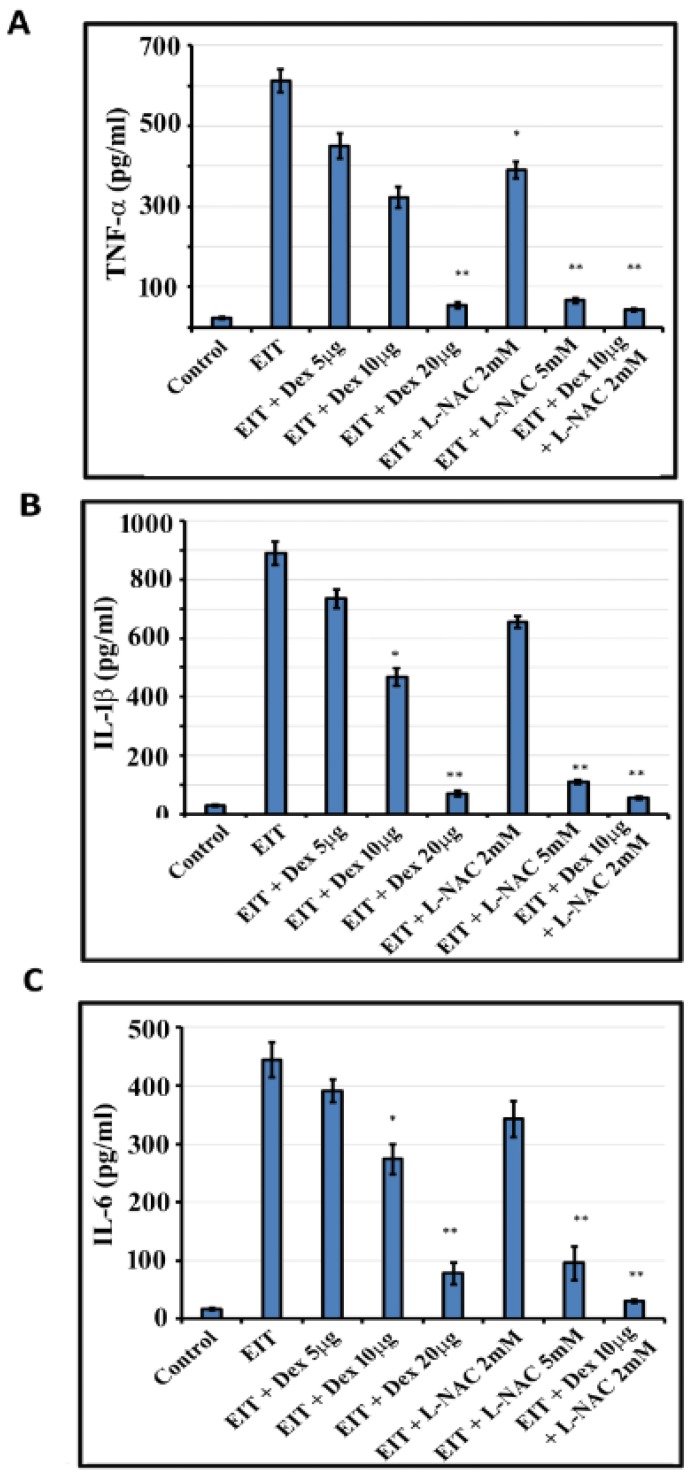
Proinflammatory cytokines. The levels of TNF-α (**A**), IL-1β (**B**), and IL-6 (**C**) were determined in OC homogenates using ELISA kits. Data are expressed as mean values ± SD and are representative of four independent experiments. * *P* < 0.01 or ** *P* < 0.001 compared to EIT group. *n* = 9 OCs per group per experiment.

OC explants exposed to EIT showed significant production of proinflammatory cytokines, TNF-α, IL-1β, and IL-6 compared with control group (*P* < 0.001) (Figure 4A–C). The higher concentrations of 20 µg/mL Dex and 5 mM L-NAC were able to decrease the production of these proinflammatory cytokines in response to EIT. However, we observed that in combination, lower concentrations of 10 µg/mL Dex + 2 mM L-NAC were able to significantly prevent the production of these proinflammatory cytokines.

### 3.4. L-NAC and Dex Attenuate NO Production

**Figure 5:**
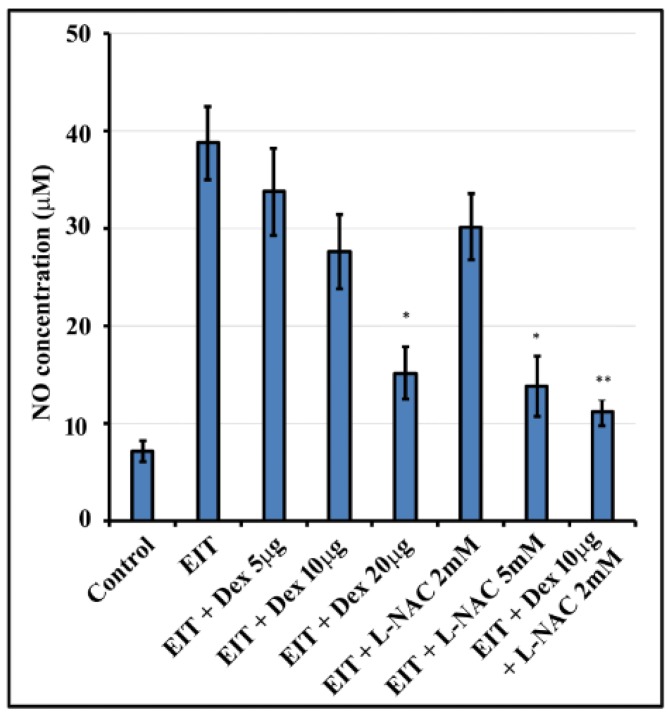
Nitric oxide (NO) levels in OC explants: NO production was determined in homogenates of OC explants. Data are expressed as mean values ± SD and are representative of three independent experiments. * *P* < 0.01 or ** *P* < 0.001 compared to EIT group. *n* = 9 OCs per group per experiment.

NO production was significantly elevated in OC explants subjected to EIT compared to control group (Figure 5). Dex and L-NAC individually were able to decrease this increase in NO production in a dose-dependent manner. Higher concentrations of Dex and L-NAC individually were able to decrease NO production. However, in combination lower doses of 10 µg/mL Dex + 2 mM L-NAC were able to significantly prevent the production of NO in OC explants exposed to EIT (*P* < 0.001).

## 4. Discussion

Despite advances in medical technologies and surgical techniques, preservation of pre-operative hearing remains a pervasive issue in implanted individuals with overall 42% having partial hearing preservation and 8% having complete loss of residual hearing [12,13,14]. This loss of residual hearing is a significant issue in expanding the indications of CI. Additionally, for severely hearing-impaired patients, while it was previously believed that the benefits of residual hearing outweigh in comparison to the restorative effects of the CI, recent advances in technology have been able to capitalize on patient’s remaining sensory capabilities. The advent of combined electric and acoustic stimulation (EAS), which supports the patient’s natural residual hearing by amplifying low-frequency sounds and high-frequency restoration via CI, has consistently outperformed CI alone [12,15]. Taken together these recent advancements and expanding patient population highlight the importance of better preserving residual hearing post-CI.

Many pathways lead to post-traumatic hair cell death and in turn residual hearing loss post-CI [6,8]. However, by large, they revolve around ROS, inflammation and upregulation of pro-apoptotic pathways [8]. The hair cell death is the result of an increase in stress-induced, pro-apoptotic molecules such as TNF-α, IL-6, IL-1β, mitogen-activated kinase (MAPK), Bax, TNFR1, and Bax/Bcl-2 ratio and caspases [9,16]. These pro-apoptotic molecules can be the result of cellular stress from several sources including inflammation, Fas-receptor activation, DNA damage, or increased endoplasmic reticulum and mitochondrial membrane permeability [17,18,19]. Nevertheless, a significant portion of inflammatory damage is due to ROS generated in key cochlear tissues [8,20].

Following trauma, large quantities of ROS and reactive nitrogen species (RNS), produced by the combination of NO with superoxide, are generated by immune cells and escape the mitochondria of compromised cells [20,21,22,23,24]. Outside of their direct oxidative damage, ROS/RNS can cause lipid peroxidation, which is a self-sustaining vasoconstrictive process that can cause local tissue ischemia. This ischemia creates the conditions to produce more ROS in hypoxic mitochondria and subsequent reproduction injury when perfusion and aerobic respiration resumes in damaged mitochondria [8,11,18]. Finally, oxidative stress can lead to inflammation and the production of pro-inflammatory and pro-apoptotic cytokines such as IL-6, IL-1β, and TNF-α [8,24]. These pro-inflammatory mediators may further propagate the redox-induced cochlear damage. Targeting these pathways presents an excellent opportunity to limit residual hearing loss post-CI. With this in mind, we propose a two-pronged pharmacological approach to neutralize ROS and inhibit key apoptotic regulatory pathways using Dex and L-NAC.

L-NAC is a free radical scavenger and a pro-drug for glutathione, the body’s primary reductive enzyme, allowing it to effectively neutralize ROS and RNS [25]. In doing so, it protects against damage to DNA, proteins, and depletion of intracellular glutathione levels thereby reducing the activation of downstream pro-apoptotic molecules MAPK/JNK, NF-κB, caspase, and Src protein kinase [9,25,26]. Indeed, L-NAC has been shown to completely prevent hearing deficits and hair cell loss in glutathione deficient mice [26]. Additionally, L-NAC’s ROS scavenging properties have consistently been shown to ameliorate cochlear damage due to acoustic-trauma at a concentration of 100 mg/kg/day as well as in manganese-induced ROS in rat pups at a concentration of 20 mM. [17,27,28]. In addition to its direct antioxidant effect, L-NAC provides protection for hair cells against TNF-α induced toxicity in OC explants [29]. Furthermore, L-NAC reduces ROS/RNS-induced glutamate excitotoxicity in spiral ganglion cell lines and can protect against both the intrinsic and extrinsic apoptotic pathways following chemical insult in hair cells and auditory nerves. Finally, L-NAC’s antioxidant effects protect against damage to vascular endothelial cells by the same mechanisms, especially by mediating NO and RNS, further limiting the influx of inflammatory mediators [30]. Our findings are in agreement with these studies demonstrating ability of L-NAC to attenuate the generation of NO and ROS that prevent damage to sensory cells [9,31].

Complementing the redox protection provided by L-NAC, Dex is a glucocorticoid with wide-ranging effects on inflammation, immune regulation, and gene transcription. Notably, in rodents, glucocorticoid receptors are found throughout the cochlea, particularly in the stria vascularis, inner hair cells, outer hair cells, and spiral ligament of the cochlea and cochlear nerve. In humans, the highest concentration of receptors is found on the spiral ligament [32,33,34]. Moreover, it has been demonstrated that the intratympanic application of dexamethasone supports cochlear homeostasis under stress conditions such as noise-trauma in multiple animal models [35,36,37]. Dex is able to modulate the immune reaction in the traumatized cochlea via NFκB, the gene pathways of cytokine receptors, and regulation of cell adhesion molecules (CAMs) [35,37].

Additionally, Dex has been demonstrated to dose-dependently protect hair cells against TNF-α-mediated apoptosis in vitro by increasing expression of anti-apoptosis genes such as PI3K/Akt, NFκB, and Bcl-2 signaling [16,38,39,40]. While simultaneously decreasing the expression of pro-apoptosis genes like MAPK/JNK, TNFR1, Bax, and production of nitric oxide, a potent vasodilator, and precursor of RNS [16,24,39,40]. Dex’s has also demonstrated significant otoprotective effects in the context of noise-trauma and cisplatin ototoxicity [41,42,43]. Moreover, this protective effect was found to be dependent upon the activation of dexamethasone-dependent classical nuclear receptor pathways [44,45].

In conjunction with its functions in the pro-apoptotic gene pathways, Dex is also implicated in indirect scavenging of ROS via the upregulation of glutathione (GSH), the body’s main reductive enzyme. Specifically, in the cochlea, Dex increases the expression of gamma-glutamylcysteine synthetase, the rate-limiting enzyme for GSH synthesis, and has been shown to decrease lipid peroxidation in the spiral ganglion [46,47]. These observations, combined with the other gene transcription effects of Dex, make it a valuable ant-inflammatory and anti-apoptotic drug.

However, because of the tightly regulated blood-labyrinth barrier, high systemic doses of Dex are necessary to achieve therapeutic concentrations in the inner ear. However, systemic Dex administration has been associated with side effects, including mood changes, immune suppression, loss of appetite, increased thirst, weight gain, hypertension, hyperglycemia, and disrupted sleep patterns. In a recent randomized clinical trial (RCT), a trans-tympanic dose of 0.5 mL of 10 mg/mL Dex 24 h prior to surgery significantly decreased pure tone threshold averages compared to oral prednisolone given 1 mg/kg/day for six days prior to surgery [48]. Although intratympanic injection of Dex dramatically reduces the dose administered, it is also associated with side effects including localized immune suppression, ion homeostasis, pain, dizziness, and the possibility of persistent tympanic perforation [49,50]. Given these undesirable side effects, it is beneficial to reduce steroid use to the minimum effective dose.

Additionally, despite its safety record, certain doses and routes of administration of L-NAC have been associated with side effects [51]. As mentioned previously, a study in rat pups showed that 20 mM L-NAC protected against Mn induced ROS [27]. Another study in guinea pigs achieved therapeutic effects with 40 mg/mL (245 mM) concentrations post-CI. However, the local delivery of L-NAC at this concentration was also associated with a transient increase in hearing thresholds and osseoneogenesis was seen in a greater number of NAC-treated guinea pigs [52].

These dosages, 0.5 mL of 10 mg/mL of trans-tympanic dex and 20 mM L-NAC, are significantly higher than the dosages in our experiments, which are approximately 5–20 µg/mL Dex and 2–5 mM L-NAC. Our goal is that these vastly smaller doses minimize their potential side effects while retaining their therapeutic effects. Our findings support the concept that trans-tympanic administration with the lowest possible therapeutic dose may offer the best combination of efficacy and safety.

In summary, our results suggest that L-NAC and Dex can be combined to lower the effective doses that can provide otoprotection for EIT. It is possible that L-NAC and Dex augment the otoprotective properties of each other. However further studies are warranted to decipher the molecular mechanisms underlying synergistic interaction between L-NAC and Dex. Further studies employing the preclinical animal models of CI will help in confirming our in vitro data. In vivo studies in different animal species such as mice, rats, and guinea pigs should be taken into account critically in future investigations. In addition, it will be worthwhile to determine whether the long-time incubation of drugs with lower concentrations show benefits in future studies. The availability of novel treatment modalities for the preservation of residual hearing will promote better clinical outcomes and improved quality of life of implanted individuals and their families.
